# Prostate adenocarcinoma presenting as diffuse osteolytic metastasis

**DOI:** 10.1002/ccr3.6685

**Published:** 2022-12-13

**Authors:** Mohanad Faisal, Abdulrahman F. Al‐Mashdali, Noor Jasim, Mohamed Salem Ismail Khalil

**Affiliations:** ^1^ Internal Medicine Department Hamad Medical Corporation Doha Qatar

**Keywords:** bone metastasis, oncology, prostate cancer, radiology

## Abstract

Prostate cancer is one of the most common cancers that metastasize to the bone. Bone metastasis is usually osteoblastic, and diffuse osteolytic lesion on presentation is unusual. Here, we are reporting MRI image of patient presented with diffuse osteolytic lesion and found to have metastatic prostate cancer.

## Abstract 2

Prostate cancer can present as diffuse osteolytic bony lesions, and clinician should keep in mind such differential when encountering such presentation.

## 1 CASE PRESENTATION

A 44‐year‐old Asian male patient presented with a 1 year history of progressive dull aching backache. It is not related to movement and is present both day and night. The patient reported 8 kg unintentional weight loss during the last 6 months. The patient reported some leg weakness also during the last few days. He denied fever and sensory symptoms with no urinary or fecal incontinence. He denied lower urinary tract symptoms. The patient is a nonsmoker and drinks alcohol on social occasions. He has no family history of cancer. The examination was unremarkable except for mild lower limb weakness due to pain in the back and lower limbs. Digital rectal examination showed flat prostate, which is hard in consistency. Laboratory workups were shown a normal complete blood count and renal function test. His serum alkaline phosphatase was 153 U/L, and serum prostate‐specific antigen was 633 ng/mL. MRI spine (FIGURE 1)revealed widespread multiple variable size osteolytic metastatic lesions in the cervical, dorsal, lumbar, and sacral vertebral bodies and a few appendicular sites showing T1 low, T2/STIR bright signal with postcontrast enhancement mainly at C5, C7, T2, T3, T6, T7, T9, T11, T12, and to, L4, S1, and S2 levels. Computed tomography of the abdomen and pelvis redemonstrated multiple spines and diffuse destructive lytic pelvic bony lesions. It showed large expansile soft tissue lesions with bony destruction involving bilateral sacroiliac joints, multiple enlarged iliac lymph nodes, and slightly enlarged non‐homogenous prostate. PET scan confirmed our findings. CT‐guided right iliac bone lesion biopsy was done, and histopathology showed metastatic adenocarcinoma of prostatic origin. IHC stains for NKX3.1 and AMACR showed that tumor cells are robust and diffusely positive and IHC stain for PSA is focally positive. The patient was diagnosed with metastatic prostate cancer, and we planned to start him on androgen deprivation therapy and antiresorptive therapy.

## 2 DISCUSSION

Prostate cancer is one of the most common cancers that manifest as bone metastasis. The nature of the bone lesion in prostate cancer is typically osteoblastic, but osteolytic bony metastasis as first presentation has been reported.^1^ It is not uncommon to see osteolytic lesion in prostate cancer as seen in one study by Cheville et al. which found that 16.4% of osseous prostatic cancer metastatic lesions were lytic but prostate cancer with first presentation of diffuse osteolytic lesion was not commonly reported.^2^ Although the pathophysiology of prostate cancer was thought to be osteoblastic, emerging evidence suggests that the development of prostate cancer bone metastases requires osteoclastic activity and osteoblastic activity. Prostate cancer cells produce a variety of pro‐osteoblastic factors that promote bone mineralization. In addition to factors that enhance bone mineralization, prostate cancer cells produce factors like the receptor activator of NFκB ligand (RANKL) that promote osteoclast activity.^3^ Clinicians and radiologists should keep in mind that prostate cancer can present with diffuse osteolytic lesions in a good proportion of patients and prostate cancer should be an important differential to be considered in such cases.

## AUTHOR CONTRIBUTIONS

**Abdulrahman Al‐Mashdali:** Writing – review and editing. **Noor Jasim:** Data curation; visualization. **Mohamed Salem Ismail Khalil:** Supervision.

## CONFLICT OF INTEREST

The authors have no conflict of interest to declare.

## CONSENT

Written informed consent was obtained from the patient for the publication of this case report.

## Data Availability

Data available upon request.
